# Evaluation of malaria microscopy diagnostic performance at private health facilities in Tanzania

**DOI:** 10.1186/s12936-019-2998-1

**Published:** 2019-11-26

**Authors:** Billy Ngasala, Samweli Bushukatale

**Affiliations:** 1 Department of Parasitology and Medical Entomology, Muhimbili University of Health and Allied Sciences, Dar es Salaam, Tanzania; 2 Department of Women’s and Children’s Health, International Maternal and Child Health (IMCH), Uppsala University, Uppsala, Sweden

**Keywords:** Microscopy, Malaria, Performance, Private health facilities

## Abstract

**Background:**

The World Health Organization (WHO) recommends use of parasitological diagnosis of malaria for all age groups in all malaria transmission settings. Many private health facilities rely on malaria microscopy for malaria diagnosis. However, quality of malaria microscopy is affected by number of factors including availability of skilled laboratory microscopists and lack of quality assurance systems in many malaria endemic countries. This study was carried out to assess quality of malaria microscopy in selected private health facilities in Tanzania.

**Methods:**

A cross sectional study was conducted from August to September, 2017. A total of 40 private health laboratories in five regions were invited to participate in the study. Data were collected by distributing standardized pre-validated malaria slide-panels to each health facility. Sensitivity, specificity, and strength of agreement (with kappa score) were calculated to assess performance in detecting and quantification of *Plasmodium* species.

**Results:**

Among the 40 health facilities, 31 (77.5%) returned their results to the reference centre (Muhimbili University of Health and Allied Sciences). Overall, the measures of malaria diagnostic accuracy were high, i.e. the sensitivity and specificity of malaria parasite detection by microscopy in the health facilities were 84.3% (95% CI 77–90) and 90.8% (95% CI 83.3–95.7), respectively. There was substantial agreement in parasite detection with (Kappa value: 0.74 (95% 0.65–0.83). However, only 17.8% (24 of 134) of blood slides were interpreted correctly at the health facilities in terms of parasite density counts.

**Conclusion:**

Although there was substantial agreement between the private health microscopists and experienced microscopists in malaria parasite detection, there was poor performance in parasite counts. This calls for regular in-service training and external quality assessments at private health facilities to enhance the skills of private health facility microscopists in malaria microscopy.

## Background

The World Health Organization (WHO) recommends use of parasitological based diagnosis prior to treatment for all cases of suspected malaria in all malaria transmission settings [1]. The advantages of confirmed diagnoses include prevention of unnecessary use of anti-malarial drugs, slow development of parasite resistance, improved case management of non-malarial fevers and monitoring of treatment outcomes [1–3].

The parasitological based tests available at the point of care in most African settings are microscopy and malaria rapid diagnostic tests (RDTs). The choice of diagnostic tool depends on local circumstances, including the skills of laboratory staff, patient case load, and epidemiology of malaria and possible use of microscopy for other diseases [4, 5]. In the majority of countries in sub-Saharan Africa (SSA) including Tanzania, the market share of anti-malarial drugs including artemisinin-based combination therapy (ACT) is in public health facilities, while the market share of malaria diagnostic tests is in private health facilities [6, 7]. Currently many private health facilities rely on microscopy especially in private for profit health facilities, while in the public health facilities, and especially in peripheral health facilities are increasingly relying on the use of RDTs [6, 7].

Microscopy of Giemsa-stained blood films has high sensitivity and specificity when used by well-trained staff, which can detect as low as 50 parasites/µL blood under field conditions [5, 8]. Other advantages of microscopy include determination of parasite densities, distinction between parasite stages, differentiation between malaria species and diagnosis of other diseases [9]. However, its accuracy and usefulness depend on the quality of the microscopes, reagents, experience of the microscopist, effective quality control and the quality assurance system [5, 9–11].

Many health facilities in developing countries fail to achieve operational standards due to lack of supervision, in service training, poor supply chains of consumables, and electricity supply [5, 10, 12, 13]. There is significant evidence of misdiagnosis and overuse of anti-malarial drugs. Some studies report 30–96% of parasite negative patients being treated for malaria [10–12]. Therefore, there is a need for training of microscopists and supportive supervision to improve malaria microscopy competencies in health facilities [4, 14–17].

Governments and other stakeholders have been working to improve quality of microscopy in public health facilities, including methods for quality accreditation of national experienced microscopists and routine validation of slide examination [4, 17–19]. There has been a sizeable effort on the part of the non-governmental organizations (NGO) community to improve malaria case management and diagnostics in Tanzania, including the work done by the MalariaCare project in partnership with the National Malaria Control Programme. This work included an external quality assurance (EQA) programme targeting both clinicians and diagnosticians in public and private facilities [15, 16].

However, increasing numbers of malaria and non-malaria fevers patients seek treatment in the private sector (private for-profit facilities, drug stores and pharmacies), where the majority of anti-malarial drugs are prescribed, and the quality of microscopy in private sector is not well documented [6, 20–22]. In SSA, an estimated 35% of febrile children receiving medicines are treated in private health facilities [23]. According to the ACTwatch survey in Tanzania, about 75% of private for profit facilities had microscopy services compared to only 25% of the public health facilities had microscopy services. Challenges of routine implementation of malaria diagnosis in the private sector includes lack of monitoring of quality, and accrediting microscopy services [24]. Hence, this study aimed to assess the accuracy of malaria microscopy at selected private health facilities in Tanzania.

## Methods

### Study design and study area

This was a cross sectional study. Quality prepared and validated Giemsa-stained malaria blood slides (positive and negative slides) collected from different donors were distributed by the Private Health Laboratory Board (PHLB) staff to the private health facilities located in the town centres of five Regions (Mara, Kilimanjaro, Ruvuma, Mbeya and Songwe Regions) from August to September, 2017. In Tanzania, there has been a significant decline in the prevalence of malaria from an average of 18.1% in 2008 to 7.3% in 2017 [25]. However, Mara and Ruvuma regions still have moderate malaria endemicity (> 10% *Plasmodium falciparum* parasite rate. Whereas Kilimanjaro, Mbeya and Songwe regions have low malaria endemicity (< 5% *P. falciparum* parasite rate [25].

In Tanzania, all private health facilities including autonomous laboratories are registered by the PHLB. The PHLB was established in 1997 and is under the Directorate of Diagnostic Services in the Ministry of Health in Tanzania. All laboratory microscopists in the surveyed health facilities were invited to participate in the study. The PHLB works in partnership with the regional/municipal, district and zonal laboratory officers in inspection and supervision of private health laboratory facilities. They have also set standard guidelines for different categories of private health laboratories which can be either autonomous or as part of a health facility.

### Sampling procedure

A total of 40 health laboratories were selected among 242 registered private health laboratories that conduct malaria microscopy in the target regions to participate in the survey. Private health laboratories were selected randomly from each region using a random number list which was generated corresponding to the number of registered private health laboratories during the survey. The number of health laboratories per region was determined using proportion to sample size. Of the 40 selected laboratories, 25 laboratories were part of health facilities i.e. health centres or hospitals and 15 were autonomous laboratory facilities.

### Sample collection and blood slide preparation

Positive blood samples were donated by ten febrile adults (aged 18 years and above) who attended at Yombo health facility in Bagamoyo District about 60 km from Dar es Salaam. Five elective students from Boston University visiting the Department of Parasitology at Muhimbili University of Health and Allied Sciences (MUHAS) volunteered to participate as negative donors. After donors provided informed consent, about 3 millilitres of whole blood were collected in EDTA tubes. Using standard operating procedures for blood slide preparations and staining, a senior microscopist at the Department of Parasitology prepared both thick and thin smears on the same slide. About 100 blood slides were made from each donor blood sample. The thin blood smears were fixed with absolute methanol. After drying, both thin and thick smears were stained for 30–45 min with 5% Giemsa working solution. Once the Giemsa-stained slides had dried, they were stored in clean, plastic slide boxes.

Two independent experienced microscopists from the Department of Parasitology, at MUHAS examined the smears for the presence/absence of malaria parasite, identification of parasite species. The parasite density per microlitre was determined from thick smear by counting asexual parasites per 200 leukocytes (or per 500 leukocytes when parasites count was < 10), by assuming a standard WBC count of 8000 leukocytes/μl. A third experienced microscopist from Ifakara Health Institute acted as an a third independent experienced microscopist in case of discordant results between the first two experienced readers. A slide was declared negative if no parasites were detected after reading 100 high power fields.

### Data collection

A total of nine slides were sent to each private health facility, including 3 high parasite density slides (range 80,000–240,000 parasites/μL), 2 low-medium density slides (range 400–36,000 parasites/μL), and 4 negative slides. They were distributed to all private health facilities in the selected Regions by PHLB inspectors. The health laboratory staff were required to examine the slides for 50 min and report whether the slides were positive or negative, and provide parasite counts per μL for the positive slides. They also administered a semi-structured questionnaire to health laboratory staff to collected information on age, sex, education status, on job training and experience in malaria microscopy. All health facilities were requested to submit their readings and filled questionnaire to MUHAS office for analysis. The Regional/District Health Laboratory Technologists and some members of District Health Management Team observed the slide reading process. Based on WHO recommendations, parasite densities were considered correct when falling between 25% ± of the mean calculated densities determined by the experienced readers.

### Data management and analysis

Data were entered into Excel 2010 (Microsoft, Seattle, WA, USA). The. Analysis was performed with STATA version 13 (Stata-Corp, College Station, Texas). Data are presented as frequencies and proportions with corresponding 95% confidence intervals (CIs). The Stata command ‘diagt’ was used to calculate sensitivities and specificities. Sensitivity was defined as the proportion of positive slides correctly read as positive, and specificity was defined as the proportion of negative slides correctly read as negative. The kappa statistic (or kappa coefficient) was used to assess the strength of agreement. Interpretation of kappa was as follows: < 0.20 slight agreement, 0.21–0.40 fair agreements, 0.41–0.60 moderate agreement, 0.61–0.80—substantial agreement, and 0.81–0.99—almost perfect agreement. Inter-reader agreement for facilities versus reference values was expressed as kappa (κ) with 95% confidence interval (CI) using the ‘kapci’ function in Stata. Multivariate logistic regression was performed to assess factors affecting performance of laboratory microscopists in parasite quantification. Independent variables included in the regression model included work experience, qualifications, history of recent on job training in microscopy and type of laboratory facility.

### Ethical considerations

The study was ethically reviewed and approved by the ethical clearance committee of MUHAS. Informed consent forms were signed by blood donors. To ensure confidentiality, the participants’ data were linked to a code number only.

## Results

### Characteristics of the surveyed private health facilities and microscopists

A total of 31/40 (77.5%) of surveyed private health facilities returned 253 panel slides to MUHAS office for further assessment. Twenty-one slides were damaged or broken during transportation and handling at the health facilities and 26 slides were missing. Among the surveyed health facilities, 64.5% (20 of 31) were primary health care facilities i.e. dispensaries or health centres. As shown in Table 1, a majority of surveyed laboratory microscopists were males, < 40 years, and had a certificate level education. Only 26.8% (15 of 56) of all participants had attended at least one short course in malaria microscopy (Table 1).

**Table 1:** Characteristics of surveyed private health facilities and microscopists

Characteristic	Health facility (N = 31)	
	(N = 31)	Percentage
Type of health facility		
Autonomous laboratories	9	29.0
Dispensaries	11	35.5
Health centres	9	29.0
Hospitals	2	6.5
Number of health facilities per region		
Mara	7	22.5
Ruvuma	6	19.3
Kilimanjaro	8	25.8
Mbeya	6	19.3
Songwe	5	16.1

Characteristic	Health facility (N = 31)	
	(N = 56)	Percentage
Microscopists		
Age group		
19–30	28	50.0
31–40	16	28.6
≥ 40	12	21.4
Sex		
Male	45	80.4
Female	11	19.6
Education qualification		
Certificate	34	60.7
Diploma	18	32.1
Advanced diploma/degree	4	7.2
Recent microscopy refresher training		
Yes	15	26.8
No	41	73.2

### Performance of health facilities in malaria diagnosis

Overall, measures of malaria diagnostic accuracy were high i.e., the sensitivity and specificity of microscopy detection of malaria parasites in the health facilities were 84.3% (95% CI 77–90) and 90.8% (95% CI 83.3–95.7), respectively (Table 2). The overall inter-reader agreement between health facility microscopists and experienced microscopists in parasite detection was high (87%) with κ = 0.74 (95%0.65–0.83). The sensitivities in detecting malaria parasites according to the type of health facility were also moderate to high (> 80%), however specificities tended to be higher in health centres and hospitals (Table 3). The inter-reader agreement according to the type of facility and experienced microscopist were also high (ranging 83.9 to 92.3) with kappa value ranging from 0.67 to 0.84.

**Table 2:** Overall performance of private health facilities microscopists in detection of malaria parasites compared to the experienced microscopists

PHF readers	Experienced reader	
	Positive	Negative
Positive	113	9
Negative	21	89
Sensitivity % (95% CI)	84.3% (77–90%)	
Specificity % (95% CI)	90.8% (83.3–95.7%)	

*PHF* private health facility, *CI* confidence interval

**Table 3:** Evaluation of the performance of microscopists in detection of malaria parasites according to the type of health facility

Type of health facility	Number of microscopists	Sensitivity %	Specificity %	Agreement	Kappa value	
		% 95% CI	% 95% CI	%	ĸ	95% CI
Autonomous laboratory	9	86.7 (73.2–94.9)	88.2 (72.5–96.7)	87.3	0.74	(0.60–0.89)
Dispensary	11	81.5 (68.6–90.7)	87.9 (71.8–96.6)	83.9	0.67	(0.51–0.83)
Health centre	9	86.2 (68.3–96.1)	95.8 (78.9–99.9)	90.6	0.81	(0.66–0.97)
Hospital	2	83.3 (35.9–99.6)	100 (59–100)	92.3	0.84	(0.60–1.00)

*CI* confidence interval

Only 24/134 (17.8%) of parasite positive blood slides were interpreted correctly at the health facilities in terms of parasite density counts (i.e., within 25% ± of the mean expert determined parasite density). There was wide variation in parasite counts at health facilities compared to the experienced microscopists, Table 4. Overall, over 50% of panel slides were correctly classified as low -medium parasite density slides whereas less than 50% of high-density panel slides were correctly classified as high parasite density slides. At the hospital level, all high density slides were correctly classified, but the two low-medium density slides were incorrectly classified as high parasite density or as being negative slides (Table 4). In the multivariate logistic regression model, only one factor; on job training in malaria microscopy was significantly associated with correct quantification of malaria parasites (Table 5).

**Table 4:** Classification of parasite quantification at different levels of private health laboratories compared to parasite density panel slides readings at MUHAS

Microscopists’ results	Low-medium density panel slides		High density panel slides	
	Slides (n)	%	Slides (n)	%
All facilities	(N = 54)		(N = 80)	
Low-medium	30	55.5^a^	37	46.3
High	9	16.7	37	46.3^b^
Negative	15	27.8	6	7.5
Autonomous laboratory	(N = 23)		(N = 31)	
Low-medium	15	65.2^a^	13)	41.9
High	3	13.0	13	41.9^b^
Negative	5	21.7	5	16.1
Dispensary	(N = 19)		(N = 26)	
Low-medium	10	52.6^a^	15	57.7
High	3	15.8	11	42.3^b^
Negative	6	31.6	0	0
Health centre	(N = 10)		(N = 19)	
Low-medium	5	50^a^	9	47.4
High	2	20	9	47.4^b^
Negative	3	30	1	5.2
Hospital	(N = 2)		(N = 4)	
Low-medium	0	0^a^	0	
High	1	50	4	100^b^
Negative	1	50	0	

^a^Correctly classified as low to medium parasite density slide

^b^Correctly classified as High parasite density slide

**Table 5:** Factors associated with correct performance in quantification of malaria parasites by microscopists in selected private health laboratories in Tanzania

Variable name	Univariate			Multivariate		
	OR	95% CI	*P*-value	aOR	95% CI	*P*-value
Qualification						
Certificate	1			1		
Diploma	0.338	0.075–1.525	0.158	1.239	0.196–7.835	0.82
Adv. diploma	0.754	0.166–3.3430	0.716	1.668	0.242–11.488	0.602
Experience (years)						
0–2	1			1		
2–5	0.5	0.104–2. 403	0.387	0.472	0.073–3.056	0.431
> 5	1.103	0.277–4.39	0.889	1.089	0.151–7.862	0.932
Onjob training (yes)	4.47	1.772–11.275	0.002	8.04	2.197–29.425	0.002*
Type of lab						
Dispensary	1			1		
Autonomous lab	0.811	0.281–2.234	0.697	0.344	0.793–1.496	0.155
Health centre/hospital	1.1	0.375–3.226	0.862	1.339	0.372–4.829	0.655

*OR* crude odds ratio, *aOR* adjusted odds ratio, *CI* confidence interval

* Significant at P-value < 0.05

## Discussion

The present study shows that there is substantial agreement in detection of malaria parasite between the private health facility microscopists and experienced microscopists at the reference centre. The majority of microscopists were able to detect malaria parasites from quality prepared blood slides. However, < 20% of the slides at the health facilities had correct parasite counts when compared with the experienced microscopists. These findings are consistent with other studies in public health facilities in malaria endemic countries [18, 19, 26, 27].

The sensitivity of microscopy is the most important parameter in case management of patients due to fear that a case of malaria would go untreated and result in serious complications. The overall sensitivity was over 80%, which is similar to the findings reported mainly in public health facilities in Kenya and Ethiopia [18, 27]. However other studies have reported poor quality of malaria microscopy diagnosis [19, 28]. A number of factors have been associated with accurate malaria microscopy diagnosis including in service training, good optical condition of microscopes, quality staining of slides and quality of smearing [18, 27, 28]. This parameter has also shown to vary according to the parasite densities [11, 29].

In this era of artemisinin-based combination therapy (ACT), specificity of a diagnostic test is also very important parameter to preserve drug supply and efficacy by decreasing the exposure of parasite populations to anti-malarial drugs. Over exposure may otherwise spur the selection of drug resistance, which is a growing major concern. The current study showed a specificity of 90.8%, which is lower than the 97% reported in a study done in Bahir Dar city administration, Northwest Ethiopia [18]. The specificity in microscopy may be influenced by the time spent in reading the slides and experience of laboratory microscopists and the lack of in-service training [18, 27].

Measurement of parasite density is also very important in clinical management of the patients, especially in patients with severe malaria. In management of severe malaria, there is a need to assess the parasite density to monitor treatment outcomes [1, 30]. Low parasite counts are more likely to result in false negative results [31, 32]. A number of studies have shown that inter-observer agreement between two skilled/experienced readers is high at high parasite densities and low at low at low parasite densities [31–35]. Although positive blood slides were collected from patients with relatively high parasite densities, majority of the private health facility microscopists had incorrect parasite quantifications compared with the readings of experienced microscopists. Hence, in facilities with limited capacity to conduct quality-assured microscopy, patients with suspected severe malaria should be refereed to accredited facilities for microscopy testing [30].

A number of factors may affect quantification performance of the private health microscopists, in this study there was significant association between the private health microscopists’ performance and recent report of on job training. Other quality assurance assessment studies have shown that recent in-service training improved the quality of microscopy [19, 26, 27, 36]. Also, discrepancies in parasite densities could be affected by mean density and random chance in the selection of fields to examine may play a large part in the between readers discrepancy, especially with low density parasitaemia slides [33, 37].

Availability and quality of diagnostic services for malaria at the private health facilities is not well documented, National governments and development partners should also direct their efforts to improve quality of malaria microscopy at the private sector [7, 21]. More efforts are required to strengthen the capacity of health workers in both public and private sectors. Studies have shown that this can be achieved through on job training programs, supportive supervision, and reliable supplies of quality reagents, electricity and establishment of an external quality assurance scheme [4, 9, 13, 15–17, 36, 38].

This study has a number of limitations, including few private health laboratories being surveyed due to limited resources. It was not possible to control for group efforts or for time limits on slide readings since supervision of slide readings was done by district health officials. There were no low-density slides in the proficiency test panels. Also some of the surveyed facilities (> 20%) did not return their results to PHLB/MUHAS and some slides were broken/damaged during the process of transportation. Furthermore, the species of malaria parasites used for malaria blood smears panel preparation were not characterized by molecular methods, such as polymerase chain reaction. The accuracy of microscopists results could have been different if the microscopists were reading slides they had made themselves since quality of staining and smear preparation could have effects on the quality of microscopy [27].

## Conclusion

The present study showed substantial performance of microscopists at the private health facilities in detection of *P. falciparum* parasites. This study also observed very poor agreement in parasite counts between readings done at the private health facilities with that of experienced microscopists. Hence, improved quality of malaria microscopy is needed at the private health facilities in order to reduce over prescription of anti-malarial drugs and for prompt and effective management of non-malaria fevers. Participation in malaria microscopy quality assurance training may help to improve performance of microscopists in the detection and quantification of malaria parasites.

## Data Availability

The datasets used and/or analyzed during the current study are available from the corresponding author on reasonable request.

## Abbreviations

ACT: artemisinin-based combination therapy
aOR: adjusted odds ratio
CI: confidence interval
EDTA: ethylenediaminetetraacetic acid
EQA: external quality assurance
MUHAS: Muhimbili University of Health and Allied Sciences
RDT: malaria rapid diagnostic test
NGO: Non Governmental Organization
OR: odds ratio
PHLB: Private Health Laboratory Board
SSA: Sub Saharan Africa
WBC: white blood cells
WHO: World Health Organization
